# BRinging the Diabetes Prevention Program to GEriatric Populations in Spanish (BRIDGE-S): Assessing Acceptability Among Hispanic/Latino Adults in an Older Adult Center

**DOI:** 10.1177/21501319261439551

**Published:** 2026-05-27

**Authors:** Elaine De Leon, Kenny Decaro, Franze De La Calle, Angieleana Soto, Angel David Santiago, Wendy Aragon, Jeannette M. Beasley

**Affiliations:** 1 NYU Grossman School of Medicine, New York, USA; 2 NYU School of Global Public Health, New York, USA; 3 NYU Steinhardt School of Culture, Education, and Human Development, New York, USA; 4 Ponce Health Sciences University School of Medicine, Puerto Rico; 5 University of Puerto Rico School of Medicine, San Juan, Puerto Rico

**Keywords:** diabetes prevention, older adult, older adult center, Spanish language, risk reduction, culturally appropriate intervention

## Abstract

**Background:**

Although the CDC’s Diabetes Prevention Program (DPP) is effective among Hispanic populations, its acceptability among older Hispanic adults, particularly in community-based settings, remains understudied.

**Methods:**

We assessed the acceptability of DPP content among Spanish-speaking Hispanic/Latino older adults attending an Older Adult Center (OAC) in a large metropolitan area. Between August 2023 and October 2024, participants attended 4 interactive, in-person DPP sessions delivered by native Spanish-speaking physicians. Post-session surveys assessed acceptability, and 2 focus groups explored participant experiences. Quantitative data were analyzed descriptively, and qualitative data underwent thematic analysis guided by the Theoretical Framework of Acceptability.

**Results:**

Sessions averaged 15 participants (77% female; *M*_age_: 71 years), representing diverse Hispanic origins, primarily Ecuadorian, Mexican, and Puerto Rican. Most reported chronic conditions including hyperlipidemia, hypertension, and type 2 diabetes. Participants described the content as culturally appropriate, understandable, and relevant. Focus groups revealed strong receptivity to lifestyle-focused content, a preference for in-person OAC-based delivery over virtual formats, and interest in additional topics such as medication side effects, natural remedies, and social determinants of diet.

**Conclusions:**

Community-based, culturally-tailored DPP session delivery at OACs was perceived as acceptable and engaging among older Hispanic/Latino adults, supporting this setting as a promising platform for delivering diabetes prevention education in this population.

## Background

Evidence from the CDC’s National Diabetes Prevention Program (DPP) demonstrates that culturally tailored lifestyle programs can reduce diabetes risk among Hispanic populations.^1,2^ However, disparities in DPP engagement exist between the general Hispanic population and non-Hispanic Whites,^1,3^ which may be further pronounced in older Hispanic adults due to additional linguistic and cultural barriers. While cultural adaptations have shown promise in enhancing DPP outcomes for Hispanic participants,^1^ there is a notable dearth of specific data regarding its effectiveness in older Hispanic adults and the potential impact of different settings on DPP engagement within this demographic.

Growing literature highlights the potential of Older Adult Centers (OACs) in fostering health and well-being among older adults through the integration of health promotion programs within these settings.^4-7^ However, differences in sociodemographic factors (age, sex, education, and income), health and well-being, accessibility, and level of social interaction have been identified as contributors to variability in OAC participation.^8^ These differences in OAC participation may hinder the reach of health interventions delivered through OACs. Despite these challenges, OACs remain promising environments for disseminating health-related information to older adults, and we hypothesized that this setting could enhance the acceptability of behavioral interventions that focus on lifestyle modification, like the DPP, among older Hispanic and/or Latino adults. As part of a quality improvement initiative conducted in partnership with an OAC in a large metropolitan area, this study aimed to assess the acceptability of delivering Spanish-language, DPP-based lifestyle modification sessions to older Hispanic and/or Latino adults in this setting.

## Methods

### Study Setting

This study was conducted at an OAC affiliated with a federally qualified health center network and an academic medical center in a large metropolitan area. The OAC is open to any city resident aged 60 years and older, with a mission to support aging adults and their caregivers through daily recreational, educational, and health promotion activities. The DPP sessions were delivered as part of the OAC’s usual health-related programming.

### Participants

Participation in the DPP sessions was open to all Spanish-speaking OAC members aged 65 years and older. Recruitment occurred through announcements in the OAC activity calendar and verbal invitations by OAC staff to all eligible members. Participation was voluntary, and participants were informed that their decision to participate would not affect access to OAC services. OAC members were not required to enroll in advance or commit to the full series; attendance was open, and individuals could join any of the scheduled sessions. No personally identifiable information was collected during sessions; only age was recorded to confirm eligibility for inclusion in the study analysis. Participants were asked not to share identifying details during sessions and data collection activities.

### Program

The program consisted of 4 in-person, group-based DPP sessions delivered at the OAC: “Managing Triggers,” “Eating Well While Out,” “Buying and Cooking to Prevent Type 2 Diabetes,” and “Understanding Energy Balance (Energy In, Energy Out).” Each session was designed to last approximately 1 h and included approximately 40 min of didactic content that was largely focused on practical strategies to support lifestyle modifications and 20 min of group discussion regarding personal experiences with session topics. Although the DPP is originally intended to encourage health behavior changes for individuals with prediabetes and prevent or delay the onset of type 2 diabetes mellitus (T2DM), all eligible members of the OAC were welcome, regardless of prediabetes status. The content was adapted and delivered in Spanish for this setting.

### Data Collection

At the conclusion of each session, all attendees were invited to voluntarily complete post-DPP session surveys. Survey questions were based on existing implementation measures^9^ used by a prior study adapting the DPP program for older adults.^10^ Table 1 shows an overview of the post-survey measures.

**Table 1.** Overview of Post-Session Survey Measures.

DOMAIN	CONSTRUCT ASSESSED	EXAMPLE ITEMS/RESPONSE OPTIONS
PARTICIPANT CHARACTERISTICS	Gender	Male, Female, Other
	Country of origin (optional)	Self-identified Hispanic/Latino country; Other (free text)
	Health conditions	Self-reported chronic conditions
DIABETES KNOWLEDGE AND PERCEPTIONS	Prediabetes knowledge	Understanding of prediabetes (Yes/No/Unsure)
	Perceived risk	Perceived risk related to Hispanic/Latino identity
SESSION EVALUATION	Overall satisfaction	“I liked the group session”
	Perceived usefulness	“The information will help me make better decisions”
	Linguistic adaptation	“Vocabulary was easy to understand and culturally representative”
	Cultural relevance	“Information was applicable to cultural heritage”
	Self-efficacy	“I feel better prepared to reach health goals”
	Behavioral intention	“I have the intention to apply the information”
PROGRAM COMPONENTS	Relevance and usefulness	Videos, discussions, guide, goal setting
NUTRITION AND PHYSICAL ACTIVITY KNOWLEDGE	Knowledge assessment	“Is it important to know the intensity and duration of physical activity?” “Do you read food labels” (Yes/No/Unsure items

These surveys collected demographic information (age, gender, country of origin, and medical history) and feedback using Likert-scale items. The surveys assessed perceptions of session usefulness, linguistic adaptation, cultural relevance, and overall satisfaction.

Following the fourth session, attendees were additionally invited to participate in focus groups conducted immediately afterward. The focus groups aimed to gather in-depth insights into participants’ perceptions of the DPP sessions, including cultural relevance, content delivery, and overall acceptability. Discussions were facilitated in Spanish using a semi-structured interview guide developed based on the TFA constructs.

Participants who volunteered for the surveys and/or focus groups received small non-monetary tokens of appreciation (eg, notebooks, tote bags, or water bottles). Survey data were stored in a spreadsheet accessible only to study team members via a secure shared drive. Focus group recordings were also stored on the shared drive, transcribed *verbatim*, translated into English, and back-translated by bilingual study team members.

### Ethical Considerations

This project was conducted as a quality improvement initiative to enhance health programming at the OAC with content based on an evidence-based lifestyle modification intervention. As this work was designed to evaluate the program rather than the participants, involved minimal risk, and did not include collection of identifiable private information, formal ethical review and written informed consent were waived. All data, including audio recordings, were securely stored on institutional servers and analyzed in aggregate.

### Theoretical Framework

This study was informed by the Theoretical Framework of Acceptability (TFA) developed by Sekhon et al,^11^ which offers a structured approach to understanding and assessing the acceptability of healthcare interventions. It identifies 7 constructs—Affective Attitude, Burden, Ethicality, Intervention Coherence, Opportunity Costs, Perceived Effectiveness, and Self-efficacy—each contributing to overall acceptability of an intervention. The TFA facilitates a systematic evaluation of stakeholders’ perspectives and experiences, helping to identify factors influencing intervention acceptability.

### Analysis

Quantitative analysis involved calculating means and ranges of demographic characteristics and Likert-scale responses to assess participant satisfaction and perceived utility of various DPP session objectives. Qualitative data were analyzed using thematic analysis, with 2 native Spanish-speaking researchers, trained in qualitative methods, independently coding the transcripts using Dedoose version 10.0.35. The deductive coding process was guided by the constructs of the Theoretical Framework of Acceptability (TFA) to identify themes related to the acceptability of the DPP sessions. Discrepancies between coders were discussed and reconciled to ensure consistency and reliability in the coding process. Primary themes were identified and elaborated upon to explore the sessions’ acceptability, and representative quotes from participants were selected to illustrate each TFA construct when available, to provide a comprehensive understanding of participants’ experiences and perceptions. To enhance the trustworthiness and rigor of the qualitative analysis, data from focus groups were triangulated with quantitative survey data to corroborate findings.

## Results

### Surveys

The sessions attracted an average of 15 participants per session, with a range of 9 to 21 individuals attending each session. Approximately half of the participants were repeat attendees, with 30% of participants in session 2, 73% in session 3, and 57% in session 4 having reported participation in at least one other previous session (23 out of a total of 48 attendees [48%]). Participants were predominantly female (77%; range: 76%-92%), with a *M*_age_ of 70.8 years (range: 65-86 years). The participants represented diverse Hispanic origins, with the most common being Ecuador (range: 16%-42%), Mexico (range: 10%-21%), and Puerto Rico (range: 11%-26%). Notably, 2 participants in Session 1 self-identified as being of Brazilian descent but spoke Spanish. They were included in the analysis because eligibility to attend the sessions was based on being a Spanish-speaking OAC member, and country of origin from a Spanish-speaking nation was not a pre-specified requirement.

A substantial proportion of participants reported pre-existing chronic conditions with a mean of 1.86 comorbidities per participant (SD = 1.83), including hyperlipidemia (range: 25%-62%), hypertension (range: 25%-48%), prediabetes (range: 18%-33%), and T2DM (range: 25%-29%; Table 2).

**Table 2.** Demographic Characteristics of Program Attendees.

Demographic characteristics	Session 1	Session 2	Session 3	Session 4
	N (%)	N (%)	N (%)	N (%)
Sex				
Male	2 (11.8)	5 (23.8)	4 (50.0)	1 (8.3)
Female	15 (88.2)	17 (81.0)	4 (50.0)	11 (91.7)
Age				
Mean (SD)	71.6 (9.6)	73.0 (6.0)	67.2 (8.1)	68.5 (9.3)
Hispanic Origin				
Guatemala	1 (5.9)	0 (0.0)	0 (0.0)	0 (0.0)
Honduras	0 (0.0)	2 (9.5)	1 (12.5)	0 (0.0)
Brazil	2 (11.8)	0 (0.0)	0 (0.0)	0 (0.0)
Colombia	0 (0.0)	3 (14.3)	0 (0.0)	0 (0.0)
El Salvador	1 (5.9)	1 (4.8)	1 (12.5)	1 (8.3)
Mexico	4 (23.5)	2 (9.5)	2 (25.0)	2 (16.7)
Puerto Rico	5 (29.4)	4 (19.0)	1 (12.5)	3 (25.0)
Ecuador	3 (17.6)	5 (23.8)	2 (25.0)	5 (41.7)
Not Reported	0 (0.0)	0 (0.0)	1 (12.5)	0 (0.0)
Total Comorbidities				
Mean (SD)	1.41 (1.54)	2.18 (1.79)	2.62 (3.11)	1.42 (0.90)
Comorbidities				
Renal Disease	0 (0.0)	0 (0.0)	1 (12.5)	1 (8.3)
Cancer	1 (5.9)	0 (0.0)	1 (12.5)	0 (0.0)
Autoimmune Condition	1 (5.9)	2 (9.5)	1 (12.5)	0 (0.0)
Liver Disease	0 (0.0)	2 (9.5)	1 (12.5)	1 (8.3)
Obesity	1 (5.9)	0 (0.0)	1 (12.5)	1 (8.3)
Respiratory Disease	2 (11.8)	5 (23.8)	2 (25.0)	1 (8.3)
Cardiac Disease	2 (11.8)	4 (19.0)	3 (37.5)	0 (0.0)
Prediabetes	3 (17.6)	7 (33.3)	2 (25.0)	4 (33.3)
Diabetes Mellitus	5 (29.4)	6 (28.6)	2 (25.0)	3 (25.0)
Hypertension	4 (23.5)	10 (47.6)	2 (25.0)	3 (25.0)
Cholesterol	5 (29.4)	12 (57.1)	5 (62.5)	3 (25.0)

Responses reflect surveys completed voluntarily, thus may not fully reflect all attendees.

Attendance and item-level response varied by session; therefore, totals may not align across demographic characteristics.

**Figure 1.**
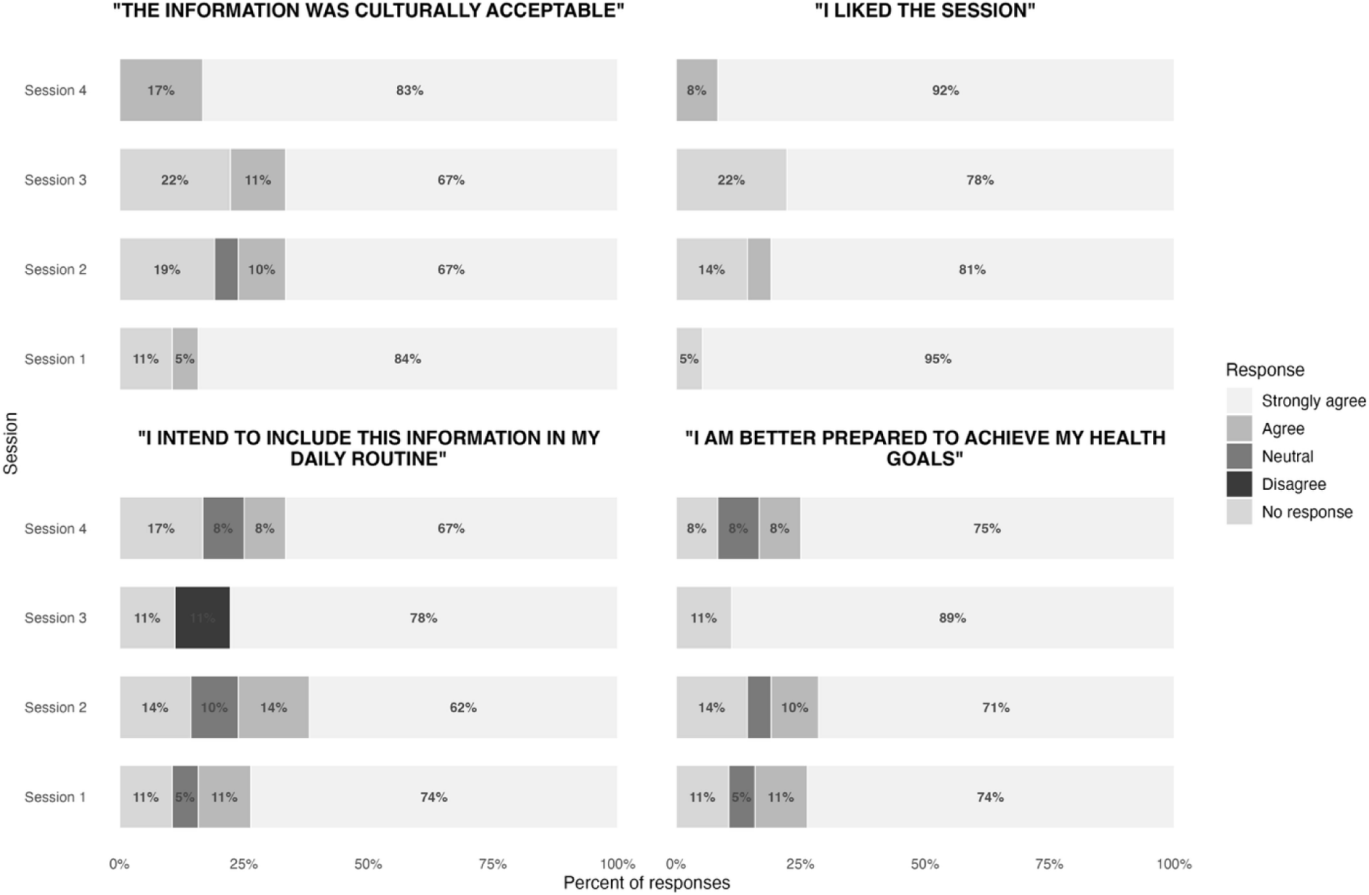
Participant Likert scale responses across 4 sessions. Responses reflect surveys completed voluntarily, thus may not fully reflect all attendees. Responses were collected using a Likert scale ranging from strongly agree to strongly disagree. The category “strongly disagree” is not shown in the figure because no respondents selected this option. “No response” represents missing responses.

Surveys indicated positive perceptions of the sessions. The content was reported to be culturally appropriate, enjoyable, and delivered with familiar vocabulary (Figure 1). Moreover, most participant responses expressed an intention to use the information presented in their daily routines and that following the sessions they felt better prepared to achieve their health goals (Figure 1).

### Focus Groups

Two Spanish-language focus groups were conducted with 18 participants (8-10 per group) following the conclusion of the fourth session. Findings from our focus group discussions, with analysis guided by the Theoretical Framework of Acceptability (TFA), revealed the degree of acceptability of the intervention among Spanish-speaking older adults (Table 3). Participants expressed positive attitudes and appreciation for the sessions. The burden of participation was alleviated by the accessibility of the intervention at the OAC, where sessions were conducted free of charge and in Spanish. Participants with medical conditions discussed previous strategies for managing them, reflecting the complex health needs of the population. They additionally shared how the lifestyle modification content they had received through the sessions felt complementary to previous health education they had received.

**Table 3.** Theoretical Framework of Acceptability Constructs With Representative Quotes From Focus Groups.

TFA construct	Construct definition	Representative quote(s)
Affective attitude	How an individual feels about an intervention	“It seems to me that with a little bit of information that you give us for us, we at least have to be grateful for it, take advantage of it, and transmit it as well. Because that information is good, but not only for us, but there are other people who also need it. Putting it into practice” (FG1) “I thought everything was very good. The information, everything was good. I have no complaints about anything. He talked about a problem and how to solve it.” (FG2)
Burden	The perceived amount of effort that is required to participate in the intervention	“Through the phone, if they are giving me class, if a fly passes me, I look where the fly is . . . in person, with you, it is more interesting. And also, I live alone. So by participating, I participate with other people. They know me, they know how I think, and I know other people and their way of thinking . . . which is also a way to relieve the emotional part.” (FG1) “. . . sometimes you’re sitting there and you’re watching what they’re doing. Maybe because of embarrassment, you don’t want to get up to do the exercise; you might do it wrong and there are other people who are watching” (FG2)
Ethicality	The extent to which the intervention has good fit with an individual’s value system	“Moderator: This program was translated into Spanish and we want to know how close it was to Hispanic culture. Participant: Very close. Another Participant: Yes.” (FG1) “Participant: Yes, it was relevant to our culture. Another participant: It was relevant, yes.” (FG2)
Intervention coherence	The extent to which the participant understands the intervention and how it works	“Well, look at the word pre-diabetes.” Pre means before diabetes. So we must know how to avoid diabetes, before diabetes is present.” (FG1 “Eat healthier, less carbohydrates and try to limit calories too. Because if you gain weight, you also get diabetes, the risk is higher. So, if you lose weight, you are healthier.” (FG2)
Opportunity costs	The extent to which benefits, profits, or values must be given up to engage in the intervention	*Not reflected in dataset.*
Perceived effectiveness	The extent to which the intervention is perceived as likely to achieve its purpose	“For example, the last time I took the class helped me because now I no longer do what I did. Sitting down to eat while watching television and everything he said. Now I have completely changed everything and I feel much better.” (FG1) “I have been to three sessions and I have put into practice many of the things that have been heard here to prevent. I don’t have diabetes but to prevent it, to learn to eat, to exercise, to know that I can’t sit in front of the television for so long without activity. I try to walk. In other words, it has helped me apply certain things that I have heard here in the sessions.” (FG2)
Self-efficac*y*	The participant’s confidence that they can perform the behavior(s) required to participate in the intervention	*Not reflected in the dataset.*

The purpose of the intervention, which was to encourage healthy lifestyles in older Hispanic adults through education on lifestyle modification, was largely understood by participants. They demonstrated comprehension of the importance of weight loss through dietary changes and exercise, albeit with some confusion regarding clinical concepts. For example, 1 participant conflated Type 1 Diabetes Mellitus with T2DM, indicating a gap in knowledge about diabetes types. Participants described the intervention as acceptable and engaging, noting that the information felt helpful and motivating and that they were open to trying behavior changes. Though some comments suggested general self-efficacy in managing overall health, self-efficacy regarding enacting program recommendations was not emphasized by participants. Importantly, sessions were deemed culturally relevant and appropriate by participants, with the use of familiar vocabulary.

Suggestions for improvement included requesting louder volume for the moderator, coverage of additional medical conditions, medication side effects, and home remedies, as well as translated sessions that would allow for inclusion of English speakers. Future versions of this program should be tailored to address the etiologies of Type 1 and T2DM and clarify how lifestyle changes primarily prevent and help manage T2DM. Participants expressed a strong preference for in-person sessions at the OAC, compared to virtually delivered sessions, highlighting the importance of community connection and support in their health journeys.

## Discussion

In this study, we utilized the Theoretical Framework of Acceptability (TFA) to assess the delivery of 4 DPP sessions aimed at encouraging healthy lifestyle modification practices among Spanish-speaking older adults affiliated with an OAC in a metropolitan area. Our findings indicate positive, affective attitudes toward the DPP sessions.

Findings from surveys and focus groups underscored participants’ receptivity toward lifestyle intervention programming, regardless of whether patients carried a diagnosis of prediabetes or other health conditions. Participants additionally provided recommendations for future sessions, including topics like medication side effects and natural remedies. Importantly, there was also a strong preference for receiving this health-related information within the familiar environment of the OAC. This highlights OACs’ crucial role in delivering culturally tailored health education to older adults.

These findings should be considered within the broader context of diabetes burden. T2DM accounts for approximately 96% of diabetes cases worldwide and ranks among the top 10 causes of death globally^12^ Community-based interventions that are acceptable to participants represent a critical component of public health efforts to address this burden, particularly among populations experiencing structural and social barriers to care such as limited English proficiency.^13^ Although effectiveness was not evaluated in this study, the high level of acceptability observed suggests that OAC-based delivery may represent a feasible platform for expanding access to prevention programming among older Hispanic and/or Latino adults. Our findings align with prior research demonstrating the value of community-based and culturally tailored delivery of the Diabetes Prevention Program (DPP), particularly in underserved Hispanic communities. For instance, a DPP and LOOK AHEAD adaptation led by Lindberg and colleagues (2021) implemented in a federally qualified health center (FQHC) setting titled *De Por Vida* found that Latina participants with both prediabetes and type 2 diabetes benefited from the intervention. Though the population of interest was not older adults, *De Por Vida* participants also appreciated the use of culturally and linguistically appropriate materials and the delivery of sessions in familiar, trusted community spaces.^14^ Additionally, another community-based DPP targeting the general Hispanic population found that participant engagement was closely tied to session attendance, further emphasizing the importance of accessible and welcoming program delivery models.^15^ Together, these studies support our conclusion that offering DPP sessions within OACs in Spanish and tailored to older Hispanic adults may enhance program acceptability and perceived relevance for those wishing to engage in programming on lifestyle modification for chronic disease prevention and management.

From an implementation science perspective, our findings additionally highlight a pathway that may influence real-world uptake of lifestyle interventions within OAC communities. Acceptability,^16^ perceived cultural relevance,^17^ and contextual fit^18^ are recognized as determinants of successful implementation and sustainability. The preference expressed by participants for OAC-based programming suggests that intervention setting functions as more than a logistical consideration; it may act as an engagement facilitator by reducing linguistic and social barriers to participation.^13^ These findings are relevant for real-world implementation, where feasibility and contextual fit influence program adoption.^18,19^

Participant feedback also provides practical guidance for program refinement. Requests for expanded health topics indicate that participants viewed the sessions as valuable and saw potential for broader application. These suggestions can inform iterative adaptation of the program to better align with participant priorities, thereby strengthening relevance and engagement. Integrating participants’ voices in this way enhances ecological validity and ensures that interventions remain responsive to community needs.

### Limitations and Strengths

This study provides important insights into community-based DPP sessions tailored specifically to older Hispanic adults, a population that remains underrepresented in the research literature. Key strengths include the use of a community-based setting and a mixed-methods approach, which allowed for a more comprehensive assessment of the sessions’ acceptability. However, several limitations should be noted. First, the study relied on self-reported data from a small sample size, both of which limit the generalizability of the findings. Self-reported measures may be subject to recall error, social desirability bias, or misinterpretation of questions, all of which could influence how participants described their experiences and perceptions. Additionally, voluntary participation in surveys and focus groups may have introduced sampling bias, as individuals who were more motivated or engaged may have been more likely to attend multiple sessions and participate in data collection, which could result in an overrepresentation of positive perspectives. Similarly, repeat attendance at sessions may have amplified the voices of participants who were already receptive to the intervention while underrepresenting those who attended fewer sessions or disengaged. These dynamics limit the ability to draw conclusions about the full spectrum of participant experiences, particularly those of individuals who may have encountered barriers or decreased motivation to continue participation.

Approximately 1-quarter of the sample reported a diagnosis of prediabetes, while the remainder reported a variety of other health conditions, including diabetes. Participation was not restricted by diagnosis, in alignment with our OAC partners’ goal of making the sessions broadly accessible to all interested members. This inclusive approach reflects real-world programming delivery within OACs, but it precludes interpretation of results for any specific clinical subgroup. Future studies should consider stratified or diagnosis-specific designs to better understand how perceptions and responses may differ based on clinical characteristics. Given that the primary aim was to assess the acceptability of lifestyle modification sessions delivered in an OAC setting, we believe this study makes a meaningful contribution to the literature on health education for older Hispanic adults.

## Conclusions

Overall, our findings demonstrate the acceptability and perceived relevance of lifestyle modification interventions within the Spanish-speaking older adult community, while also identifying OACs as settings that can be leveraged to deliver health-related programming. The community-based setting encouraged participants to attend the sessions, allowing them to feel more comfortable in participating. By offering the sessions in Spanish, we were able to make the program accessible to the older Hispanic population. Those living with T2DM also described the information as appealing and relevant, suggesting that the program was acceptable across older adults with varying health conditions.

These findings emphasize the importance of cultural relevance and community-based delivery, particularly for older Hispanic adults, and can help guide the development of future iterations of this intervention. However, this study did not evaluate effectiveness or sustained behavior change. Further research is needed to explore the long-term outcomes of the DPP interventions when delivered in these settings to address disparities in long-term accessibility, outreach, and engagement in diverse populations.

## References

[1] McCurley JL Gutierrez AP Gallo LC. Diabetes prevention in U.S. Hispanic adults: a systematic review of culturally tailored interventions. Am J Prev Med. 2017;52(4):519-529. doi:10.1016/j.amepre.2016.10.028 27989451 PMC5362335

[2] Clennin MN. Weight loss disparities among Hispanic and underserved participants, Colorado, 2015–2018. Prev Chronic Dis. 2020;17:E162. doi:10.5888/pcd17.200228 PMC7785321 33357308

[3] Ritchie ND Christoe-Frazier L McFann KK Havranek EP Pereira RI. Effect of the national diabetes prevention program on weight loss for English- and Spanish-speaking Latinos. Am J Health Promot. 2018;32(3):812-815. doi:10.1177/0890117117698623 28320212

[4] Beasley JM Sevick MA Kirshner L Mangold M Chodosh J. Congregate meals: opportunities to help vulnerable older adults achieve diet and physical activity recommendations. J Frailty Aging. 2018;7(3):182-186. doi:10.14283/jfa.2018.21 30095149 PMC7605513

[5] Beasley JM Kirshner L Wylie-Rosett J Sevick MA DeLuca L Chodosh J. BRInging the Diabetes prevention program to GEriatric populations (BRIDGE): a feasibility study. Pilot Feasibility Stud. 2019;5(1):129. doi:10.1186/s40814-019-0513-7 31741744 PMC6849183

[6] Kramer MK Vanderwood KK Arena VC , et al. Evaluation of a diabetes prevention program lifestyle intervention in older adults: a randomized controlled study in three Senior/Community Centers of Varying Socioeconomic Status. Diabetes Educ. 2018;44(2):118-129. doi:10.1177/0145721718759982 29514568

[7] Leveille SG Wagner EH Davis C , et al. Preventing disability and managing chronic illness in frail older adults: a randomized trial of a community-based partnership with primary care. J Am Geriatr Soc. 1998;46(10):1191-1198. doi:10.1111/j.1532-5415.1998.tb04533.x 9777899

[8] Krout JA Cutler SJ Coward RT. Correlates of senior center participation: a national analysis. Gerontologist. 1990;30(1):72-79. doi:10.1093/geront/30.1.72 2311964

[9] Clinton-McHarg T Yoong SL Tzelepis F , et al. Psychometric properties of implementation measures for public health and community settings and mapping of constructs against the Consolidated Framework for Implementation Research: a systematic review. Implement Sci IS. 2016;11(1):148. doi:10.1186/s13012-016-0512-5 27821146 PMC5100177

[10] Beasley JM Johnston EA Costea D , et al. Adapting the diabetes prevention program for older adults: descriptive study. JMIR Form Res. 2023;7(1):e45004. doi:10.2196/45004 PMC10498315 37642989

[11] Sekhon M Cartwright M Francis JJ. Acceptability of healthcare interventions: an overview of reviews and development of a theoretical framework. BMC Health Serv Res. 2017;17(1):88. doi:10.1186/s12913-017-2031-8 28126032 PMC5267473

[12] GBD 2021 Causes of Death Collaborators. Global burden of 288 causes of death and life expectancy decomposition in 204 countries and territories and 811 subnational locations, 1990–2021: a systematic analysis for the Global Burden of Disease Study 2021. Lancet. 2024;403:2100-2132. Accessed March 9, 2026. https://www.thelancet.com/journals/lancet/article/PIIS0140-6736(24)00367-2/fulltext 38582094 10.1016/S0140-6736(24)00367-2 PMC11126520

[13] Hall DL Lattie EG McCalla JR Saab PG. Translation of the diabetes prevention program to ethnic communities in the United States. J Immigr Minor Health. 2016;18(2):479-489. doi:10.1007/s10903-015-0209-x 25910619

[14] Lindberg NM Vega-López S LeBlanc ES , et al. Lessons learned from a program to reduce diabetes risk among low-income Hispanic women in a community health clinic. Front Endocrinol. 2021;11:489882. doi:10.3389/fendo.2020.489882 PMC7821047 33488511

[15] Ockene IS Tellez TL Rosal MC , et al. Outcomes of a Latino community-based intervention for the prevention of diabetes: the Lawrence Latino diabetes prevention project. Am J Public Health. 2012;102(2):336-342. doi:10.2105/AJPH.2011.300357 22390448 PMC3483988

[16] Proctor EK Bunger AC Lengnick-Hall R , et al. Ten years of implementation outcomes research: a scoping review. Implement Sci. 2023;18(1):31. doi:10.1186/s13012-023-01286-z 37491242 PMC10367273

[17] Manson SM. The role of culture in effective intervention design, implementation, and research: its universal importance. Prev Sci. 2020;21(1):93-97. doi:10.1007/s11121-019-01065-7 31659610 PMC6980655

[18] Nilsen P Bernhardsson S. Context matters in implementation science: a scoping review of determinant frameworks that describe contextual determinants for implementation outcomes. BMC Health Serv Res. 2019;19(1):189. doi:10.1186/s12913-019-4015-3 30909897 PMC6432749

[19] Wisdom JP Chor KHB Hoagwood KE Horwitz SM. Innovation adoption: a review of theories and constructs. Adm Policy Ment Health Ment Health Serv Res. 2014;41(4):480-502. doi:10.1007/s10488-013-0486-4 PMC3894251 23549911

